# Synthesis of Metal Matrix Composites Based on CrxNiy-TiN for Additive Technology

**DOI:** 10.3390/ma14205914

**Published:** 2021-10-09

**Authors:** Alexey Matveev, Vladimir Promakhov, Nikita Schultz, Alexander Vorozhtsov

**Affiliations:** Center for Additive Technologies, National Research Tomsk State University, Lenin Avenue, 36, 634050 Tomsk, Russia; vvpromakhov@mail.ru (V.P.); schulznikita97@gmail.com (N.S.); abv1953@mail.ru (A.V.)

**Keywords:** self-propagating high-temperature synthesis, additive technologies, composite materials, phase composition, structure, mechanical properties

## Abstract

The novelty of this work consists of obtaining original fundamental data on the laws of synthesis of new metal matrix composite materials for additive technologies. CrN + TiNi composites were obtained using the method of self-propagating high-temperature synthesis. In this work, analysis of the parameters of the synthesis of composite materials as well as their structure and phase composition were carried out. A scheme for the formation of a composite structure is established; it is shown that the phase composition is represented by 54.6 wt.% CrN and 45.4 wt.% TiNi. It was shown that composites based on the system are suitable for machines that make use of direct laser deposition to grow layers of materials. Sample structure and phase parameters were studied. It is shown that titanium nitride particles are uniformly distributed in the CrNi intermetallic matrix, the TiN particle size ranges from 0.3 to 9 μm and the average particle size is 2.8 μm. The results obtained indicate the possibility of synthesizing promising metal matrix composite materials for additive technologies. Such materials may have increased hardness, operating temperature and strength.

## 1. Introduction

Thanks to its technological advantages (i.e., improved properties of finished products, significant raw material cost reduction, and the possibility of manufacturing products with complex geometry, mobility of production, and accelerated data exchange), additive manufacturing makes it possible to produce highly complex parts for various applications quickly and cost-effectively, and every year additive manufacturing integrates more deeply into modern industry [1,2,3,4,5,6]. Today, one of the main tasks of researchers in the field of additive technologies is the development of new powder materials that can be used in additive manufacturing (AM) machines [7]. A promising direction in this area is the development of powders of heat-resistant materials, since they are in demand in the engineering, nuclear, oil, automobile, and aerospace industries [8]. For example, there are alloys formed from nickel-chromium powders, and such alloys have high elevated-temperature strength and heat resistance in oxidising atmospheres (up to 1250 °C) [9,10,11]. 

A special case of alloys based on nickel and chromium is powder materials of the Inconel family, which are successfully used in additive manufacturing. Thus, in [12] the authors demonstrated the successful use of Inconel 625 powders as raw materials for fabricating items by deposition in AM machines. The authors claim that the mechanical properties of these materials exceed the minimum requirements of ASTM F3056-14 standards. It is noteworthy that ASTM F3056-14 standards form the basis for AM-produced components to meet the mechanical properties equivalent to those of forged and/or deformed nickel alloy products [13]. However, in [14,15,16], the respective authors demonstrated that the mechanical properties of materials that were obtained by additive manufacturing using powders based on nickel and chromium alloys are inferior to typical forged materials obtained from the same alloy.

To improve the physical and mechanical properties and performance characteristics of a produced item, nickel and chromium-based alloys are reinforced with ceramic inclusions [17].

In previous studies [18,19,20,21], it was shown that the presence of ceramic particles in materials based on nickel and chromium leads to an increase in the tensile strength and elastic modulus of 15–30% (compared to pure Ni-Cr alloys), both at room temperature and at higher temperatures (up to 900 °C). In addition, Fattahi et al. [22] and Cooper et al. [23] found that the presence of ceramic particles in materials based on Ni-Cr leads to an increase in their hardness of 15–40% compared to the original materials.

Meanwhile, conventional technologies for producing composites based on nickel and chromium with ceramic inclusions have a number of disadvantages. The introduction of ceramic particles into the melt during the preparation of raw materials for AM, as well as the direct addition of ceramic powders during additive manufacturing, can lead to their agglomeration, which undoubtedly reduces the physicomechanical properties of the materials obtained. Therefore, there is a need to find methods to obtain ceramic metal composites consisting of a matrix based on a nickel-chromium alloy with uniformly distributed ceramic inclusions.

One of the possible methods for obtaining powders of high elevated-temperature strength materials is self-propagating high-temperature synthesis (SHS) (i.e., combustion), which allows for obtaining various classes of compounds: carbides, borides, nitrides, silicides, oxides, intermetallic compounds, and composites [24]. The products of SHS have high elevated-temperature strength parameters (up to 1500 °C) due to the phase composition and special structure, consisting of a refractory intermetallic matrix and micro-sized particles of refractory compounds that are uniformly distributed within [25]. In addition, the SHS method stands out from other methods due to its simplicity, synthesis rates, and minimal energy consumption [26].

Promakhov et al. [27] and Zhukov et al. [28] demonstrated the successful production of Al-TiB_2_ and (Ni-Ti)-TiB_2_ composite materials and found that composites that were obtained by SHS consist of a metallic or intermetallic matrix (Al, Ni-Ti) and ceramic TiB_2_ particles whose size varies from 0.1 to 5 μm. At the same time, it was shown by Promakhov et al. [27], Zhukov et al. [29], and Matveev et al. [30] that by changing the composition of the initial mixtures as well as the synthesis conditions, it is possible to control the phase composition and structure of SHS composites, including the size of ceramic inclusions. After being processed, composite powder materials obtained by SHS can be successfully used as raw materials in additive manufacturing machines.

The purpose of this work is to study the phase composition and structure of composite powders that are obtained from a CrN-TiNi powder mixture by SHS and to study the phase composition of the structure and mechanical properties of materials obtained by additive manufacturing using these composite powders.

## 2. Materials and Methods

### 2.1. Materials and Method of Obtaining SHS Powders Used in Additive Manufacturing

**Figure 1:**
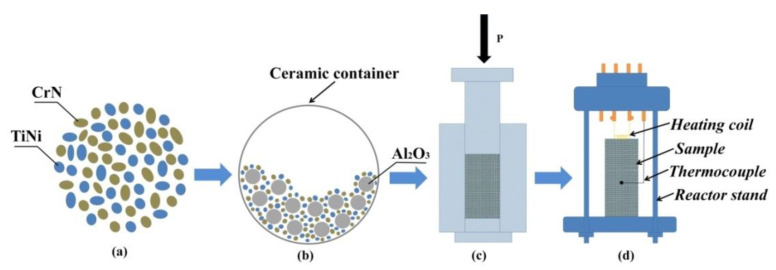
Initial scheme for the preparation of samples: (**a**) the components of the initial powder mixture, (**b**) mixing components in a ball mill, (**c**) pressing of samples from powder mixture 54.6 wt.% CrN + 45.4 wt.% TiNi, (**d**) the reactor stand with a sample obtained from a powder mixture.

CrN (average size of 50 µm, JSC “Polema”, Tula, Russia) and TiNi (average size of 70 µm, JSC “Polema”, Tula, Russia) powders were used as the initial components for producing samples of SHS composites. The initial scheme for the preparation of samples is shown in Figure 1.

CrN and TiNi powders were mixed in the stoichiometric ratio 54.6 wt.% CrN + 45.4 wt.% TiNi (Figure 1a) in a ball mill with a ceramic container and aluminium oxide balls (Figure 1b), with a mixing time of 60 min. Samples that were 23 mm in diameter and 50 mm high were prepared from the obtained mixture by cold uniaxial pressing (Figure 1c), with a pressing pressure of 23 MPa. The obtained samples were mounted on a stand (Figure 1d), which was placed in a constant pressure reactor. Then, the reactor vessel was vacuumed and filled with inert gas (argon). For different experiments, argon pressure in the reactor was 0.5 MPa, 1 MPa, and 3 MPa. The SH synthesis processes were initiated by heating the upper part of the sample with a molybdenum coil. The obtained SHS composites were ground into powder in a planetary mill. Steel balls of different diameters (from 5 to 20 mm) were selected as the grinding media, and the mass ratio of the balls to the SHS product was 1 to 1. The mill rotation frequency was 14 Hz. Further, the resulting powder was classified using sieves with different cell sizes. For the implementation of additive growing processes, a powder of a fraction of 50–150 microns was selected. It should be noted that the ground powder with particle size 50–150 microns was 80 wt.%.

### 2.2. Obtaining Samples of Composite Materials Using Additive Manufacturing

The samples were obtained from the synthesised composite powders by direct laser deposition (DLD). This method is a special case of additive manufacturing, in which there is a directed release of laser energy into powders. For direct laser deposition, the LS-3 fibre optic ytterbium laser (IRE-Polyus, Fryazino, Moskovskaya Obl., Russian Federation) was used. Laser radiation was focused using the FLW D30 process head (IPG Photonics, Oxford, MA, USA). A COAX9 coaxial weld deposition nozzle (Fraunhofer ILT, Aachen, Germany) was used to form a gas-powder jet. The LRM-200iD_7L industrial robot (Fanuc, Oshino, Yamanashi, Japan) was used as a manipulator. The ranges of the laser parameters that were used are presented in Table 1.

**Figure 2:**
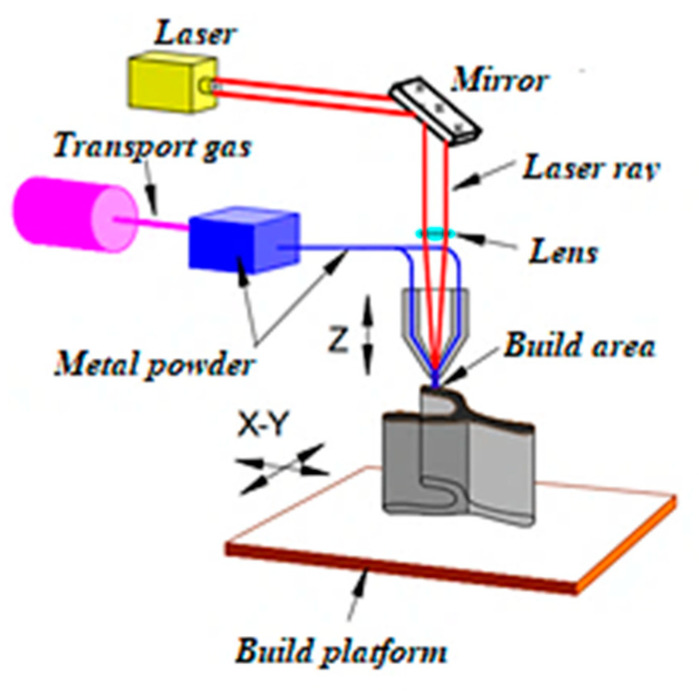
Schematic diagram of obtaining samples using direct laser deposition.

The direct laser deposition was carried out on a 7 mm thick steel substrate. The method of obtaining samples includes the supply of a gas-powder mixture directly to the growing zone. The schematic diagram and the principle of obtaining samples using DLD is shown in Figure 2.

### 2.3. Research Methods

The synthesis temperature of the materials was measured using tungsten-rhenium thermocouples inserted into the sample. The measurement results were presented in the form of thermograms on a computer. The phase composition of all the synthesis products was investigated using a Shimadzu XRD-6000 diffractometer (Cuka radiation, Ni filter) (Shimadzu Corporation, Tokyo, Japan). The phases were detected by comparing peaks and the obtained diffractograms with the Powder Diffraction File 4 database of the International Centre for Diffraction Data (ICDD^®^; PA 19073, USA). XRD analysis was performed with Shimadzu XRD-6000 X-ray diffractometer in Cu Kα-radiation (wavelength λ = 1.5406 Ǻ). The scanning range was 2θ = 10–80°, with the scanning step of 0.02°/s and acquisition time of 1 sec. Phase content, lattice parameters, and CSR size calculations were performed by structure refinement according to the full-profile analysis method (Rietveld method) [31,32]. The structure of the synthesis products was investigated on a QUANTA 200 3D scanning electron microscope with focused ion beams fitted with an energy dispersive X-ray spectrometry (EDS) 4 of 13 (Fisher Scientific, Waltham, MA, USA), necessary for studying the elemental composition of local regions of the structure of all the obtained materials. The particle size of the reaction products was measured by the line cutting method (i.e., the division of the number of intersections by the actual line length), which was applied to the SEM images that were obtained. The microhardness of the samples that were obtained by direct laser deposition was measured on a table top nanoindentation tester (NHT-TTX; CSM Instruments, Needham, MA, USA) at the maximum load of 100 mN, with the maximum indentation depth of 2100 nm, and with the loading time of 15 s.

## 3. Results and Discussion

### 3.1. Investigation of Combustion in the CrN-TiNi Mixture Samplers

Investigations of the composite synthesis processes, in the combustion mode of CrN-TiNi mixture samples at different argon pressures, showed that the synthesis of materials could not be fully completed at the argon pressure of 0.5 MPa in the reactor. The flame front extinguished immediately after the initiation of the fusion reaction. An increase in the argon pressure in the reactor to 1 MPa allowed the flame front to propagate to the middle of the sample in the spin combustion mode, after which the flame front decayed. It was not possible to measure the reaction temperature in the samples that were synthesised by SHS at the pressures of 0.5 and 1 MPa. An increase in the argon pressure in the reactor to 3.5 MPa made it possible to fully complete the SHS process in the sample. The flame front propagated in the spin combustion mode without attenuation. A similar dependence was observed in [26], where the authors studied the processes of SHS in the TiO_2_-Mg system. With a stoichiometric ratio of the components, the synthesis reaction does not proceed completely. The authors of this work explained this phenomenon as the evaporation of magnesium during synthesis, which leads to a depletion of the initial mixture and a decrease in the completeness of the reaction. An increase in the argon pressure in the reactor led to an increase in the completeness of the synthesis reaction in the system.

Based on the experimental data obtained and the results presented in [26], it can be assumed that during the synthesis, a decomposition of CrN occurs, followed by the evaporation of nitrogen into the reactor vessel at a low argon pressure. This leads to a depletion of the initial mixture, a decrease in the reaction completeness and, therefore, the attenuation of the flame front. An increase in pressure to 3.5 MPa makes it possible to prevent the release of nitrogen from the sample and to carry out a complete synthesis reaction.

**Figure 3:**
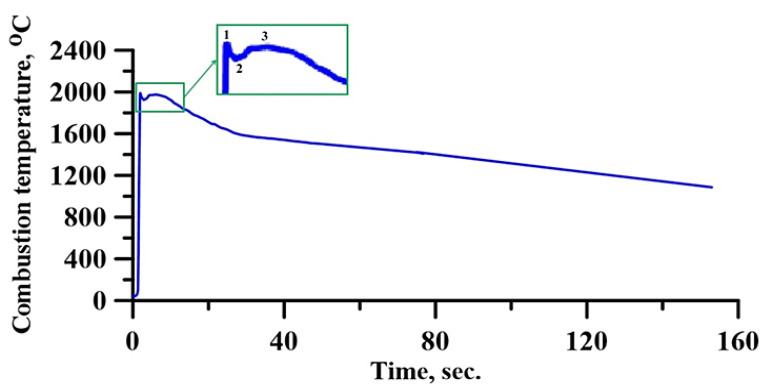
Thermogram of the synthesis of CrN-TiNi samples taking place at the argon pressure of 3 MPa.

Mossino [33] and Shekari et al. [34] found that the spin flame front arises at low heat release during the exothermic reaction of the components, as well as during endothermic processes occurring in the system. A thermogram of the synthesis of CrN-TiNi samples taking place at the argon pressure of 3 MPa is shown in Figure 3.

The peak of the thermogram (area 1) characterises an exothermic reaction (reaction with the release of heat) of the initial components taking place in the CrN-TiNi mixture sample. The reaction is accompanied by the dissipation of a great amount of heat. The apex of the peak corresponds to the system combustion temperature, which is 2000 °C. After the peak, a small decrease in the reaction temperature is observed (area 2), due to the intense heat absorption in the adjacent areas of the initial mixture. Promakhov et al. [27] explain this behaviour as the endothermic processes in the reaction zone that are associated with melting components (NiB and Ti). In addition, it was shown in [33] that chromium nitride decomposes at a temperature of 1770 °C into nitrogen and chromium. Comparing the obtained results with the data from [27,35], it can be assumed that endothermic processes occur in the CrN-TiNi samples through the incongruent melting of the CrN alloy with the evolution of nitrogen and melting of titanium. Then, temperature levelling occurs throughout the entire layer (area 3) and a further temperature decrease takes place, which is characteristic of the cooling of synthesis products.

### 3.2. Investigation of the Phase Composition and Structure of SH Synthesis Products Obtained from a CrN-TiNi Mixture

**Figure 4:**
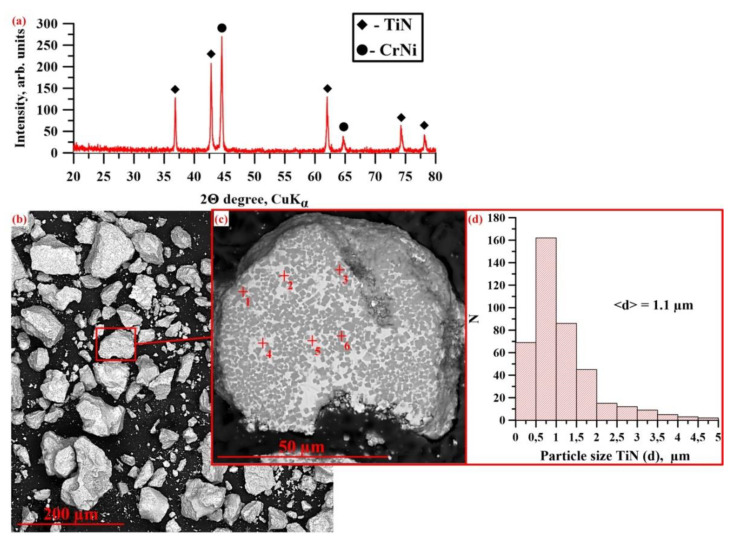
X-ray diffraction pattern of SHS powders obtained from a CrN + TiNi mixture (**a**), SEM images of these powders (**b**,**c**), and a histogram of TiN particle size distribution (**d**).

Figure 4a shows an X-ray diffraction pattern of SHS powders obtained from a CrN + TiNi mixture. The results of the X-ray diffraction analysis are presented in Table 2.

The X-ray diffraction analysis of the combustion products of the CrN-TiNi powder mixture demonstrated the presence of TiN (70 wt.%) and CrNi (30 wt.%) phases. A comparison of the lattice parameters of titanium nitride in SHS composites with the data of Newport et al. [36] and Samsonov [35] makes it possible to determine that the resulting compound corresponds to the TiN0.95 phase. Based on the results of the coherent scattering region (CSR) size measurement, it can be assumed that the crystallite size in TiN and CrNi particles is 45 nm and 43 nm, respectively. Figure 4b,c shows SEM images of fine-ground synthesis products manufactured from the CrN-TiNi mixture as well as the structure of the particles of the materials obtained. The results of the elementary analysis of the local regions of the structure shown in Figure 4 are presented in Table 3. It was found that after grinding the synthesis products, a powder was obtained whose particles have an irregular acute-angled shape and the particle size did not exceed 100 µm. Comparing the results of the elementary analysis with the data of the X-ray phase analysis, it was found that the particles of the synthesis products have a composite structure and consist of a CrNi matrix and reinforcing TiN inclusions that are uniformly distributed within it. Titanium nitride particle size varies in the range from 0.02 to 5 μm, and the average particle size is 1.1 μm (Figure 4d). In this case, the largest contribution to the particle size distribution is made by particles whose size varies in the range from 0.5 to 0.8 μm. In addition, pores with a size of up to 10 µm were found in the particles of SHS composites. The appearance of pores during synthesis is a common effect in SHS processes and is explained by the release of impurities that are present in the initial powders (O_2_, N_2_ Cl_2_, etc.).

**Figure 5:**
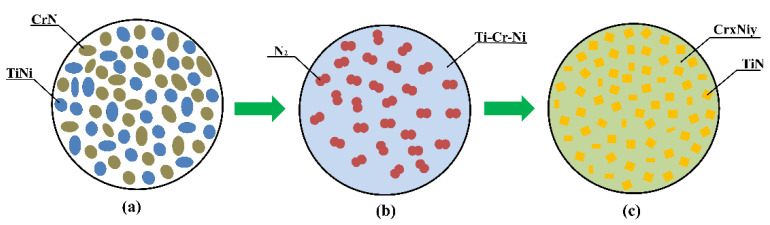
Diagram of the formation of SHS-produced CrNi-TiN materials in the process of the combustion of the CrN-TiNi powder mixture: (**a**) the initial components of the CrN-TiNi powder mixture, (**b**) decomposition and melting of components of the CrN-TiNi powder mixture, (**c**) the structure diagram of synthesis products CrNi-TiN.

In combination with the data presented in [26,27,35], the results obtained from the investigation of the combustion processes in CrN-TiNi samples and the phase composition and structure of the synthesis products allowed us to suggest a mechanism of the formation of the CrNi-TiN composite. Figure 5 presents a diagram of the formation of SHS-produced CrNi-TiN materials in the process of the combustion of the CrN-TiNi powder mixture.

After heating the upper surface of the sample with a molybdenum coil, the heat is transferred to the layer of the main initial mixture (Figure 4a) via the conduit transfer mechanism. A heating zone is formed, in which CrN decomposes with the release of nitrogen and the melting of chromium and TiNi particles, accompanied by the formation of a Cr-Ni-Ti melt (Figure 4b). Then, exothermic nitriding of titanium particles occurs with the formation of the TiN0.95 phase and the release of a large amount of heat. It is worth noting that at a pressure of less than 3.5 MPa, nitrogen is released into the reactor chamber, which leads to the depletion of the system and the termination of the synthesis reaction. It is noteworthy that a small portion of the unreacted nitrogen is presumably dissolved in the Cr-Ni melt. After cooling of the synthesis products, a composite structure is formed consisting of TiN0.95 particles, which are uniformly distributed in the CrNi matrix (Figure 4c). The heat that is released during exothermic nitriding is transferred to the next layer of the sample by conductive and convective transfer.

### 3.3. Investigation of the Phase Composition and Structure of Materials Obtained by Direct Laser Deposition from CrNi-TiN SHS Composites

**Figure 6:**
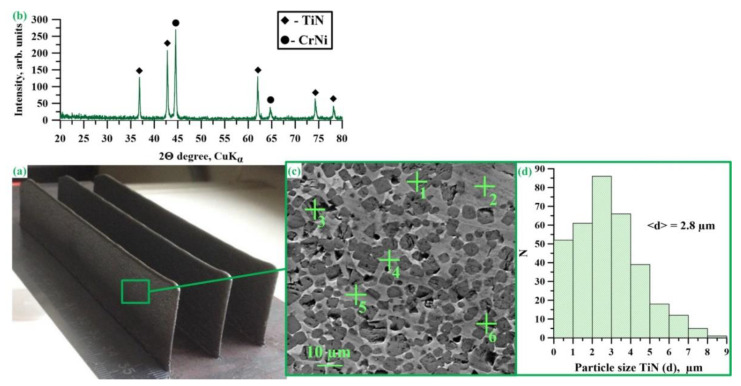
Appearance of the materials obtained by direct laser deposition from CrNi-TiN (**a**), SHS composites, an X-ray diffraction pattern and a SEM image of these materials (**b**,**c**), a histogram of TiN particle size distribution (**d**).

Figure 6a shows the appearance of materials obtained by direct laser deposition from CrNi-TiN SHS powder composites. The X-ray diffraction pattern of the materials obtained is shown in Figure 6b. The results of X-ray phase analysis are presented in Table 4. It was found that the phase composition of the obtained materials does not qualitatively differ from the phase composition of SHS composite powders and TiN phases that are present (70 wt.%), CrNi (30 wt.%). In this case, the lattice parameters of titanium nitride in the materials obtained differ from the lattice parameters in the SHS composite and are equal to 4.24 Å. A comparison of these parameters with the data from Newport et al. [36] and Samsonov [35] makes it possible to establish the obtained compound corresponding to the stoichiometric TiN1.0 phase. In addition, an increase in the size of TiN and CrNi crystallites is observed, which equals 85 nm and 55 nm, respectively. In the process of direct laser deposition, particles of the SHS composite powders melt under the influence of high temperatures produced by the laser beam. The heat released from the beam initiates the reaction by interaction of the remaining part of the nitrogen with TiN0.95 particles to form stoichiometric titanium nitride (TiN1.0), while also leading to the growth of TiN and CrNi crystallites. The structure of the materials obtained by DLD from the powders of CrNi-TiN SHS composites is shown in Figure 6c. The results of the energy dispersive analysis of the local regions in the structure of the obtained materials are presented in Table 5. It was found that the structure of the materials obtained by direct laser deposition inherits the structure of SHS composites. Titanium nitride particles are uniformly distributed in the CrNi intermetallic matrix, with particle size in the range of 0.3 and 9 µm and an average particle size of 2.8 µm (Figure 6d). Comparing the results obtained with the data on the size of titanium nitride in SHS composites, it can be assumed that the recrystallisation of titanium nitride particles occurs under the influence of high temperatures from the laser beam, which leads to an increase in their size [37,38]. In addition, in the materials obtained by direct laser deposition, pores with a size of up to 10 µm were observed as well as non-melting. The pores in the samples are inherited from the particles of SHS powders and also occur together with non-melts during the action of a laser beam. In order to optimize the structure of these materials, it is necessary to conduct a set of additional studies, which will be carried out in the next part of our work.

### 3.4. Investigation of the Mechanical Properties of Materials Obtained by Direct Laser Deposition from CrNi-TiN SHS Composites

The microhardness of the samples of materials obtained by direct laser deposition from CrNi-TiN SHS composites varied in the range of 645 to 827 HV, and the average microhardness of the samples was 760 HV. Hu et al. [39], Kaynak et al. [40], and Yang et al. [41] found that the microhardness of the samples obtained by DLD from the powders of Inconel 625/718 alloys was 250–390 HV. It can be assumed that an increase in the microhardness by a factor of two to three is achieved due to the ceramic inclusions of TiN.

## 4. Conclusions

This work demonstrated the possibility of obtaining new composite CrNi-TiN powders in the mode of self-propagating high-temperature synthesis, as well as their use as a powder raw material for additively manufacturing materials by direct laser deposition. The synthesis temperature, phase composition, and structure of SHS composites were investigated. A mechanism of their formation in the course of the reaction of the initial mixture components was proposed: 54.6 wt.% CrN + 45.4 wt.% TiNi. The phase composition, structure, and mechanical properties of the materials obtained by DLD from CrNi-TiN SHS composite powders were studied. It was found that the materials obtained inherit the phase composition and matrix structure of SHS composites. It was shown that titanium nitride particles make it possible to increase the hardness of the materials compared to Inconel 625/718 powders, by a factor of two to three. 

## Figures and Tables

**Table 1:** Parameters of the direct laser deposition facility used.

Radiation Power, W	Process Speed, mm/s	Layer Pitch, mm	Beam Diameter, mm	Powder Flow Rate, %
1300	25	0.6	2	40

**Table 2:** The results of X-ray diffraction analysis of SHS powders obtained from a CrN + TiNi mixture.

Sample of the Initial Mixture	Phases Discovered in the Synthesis Products	Phase Content, Mass %	Lattice Parameters, Ǻ	CSR Size, nm
CrN-TiNi	TiN_225	70	a = 4.2374	45
	CrNi_229	30	a = 2.8849	43

**Table 3:** Results of the energy dispersive analysis of local regions in the structure of CrNi-TiN powders produced by SHS.

Point No. (+)	Detected Elements, at. %			
	Ti	N	Cr	Ni
1	63.6	17.5	16.4	2.6
2	28.5	9.2	48.5	17.3
3	48.9	13.3	33.3	4.6
4	27.7	11.2	27.3	33.8
5	25.7	10.2	29.5	34.6
6 *	32.4	14.9	47.1	5.6

* The areas of elemental analysis in Figure 4.

**Table 4:** Results of the X-ray diffraction analysis of the materials obtained by direct laser deposition from CrxNiy-TiN SHS composites.

Sample	Detected Phases	Phase Content, Mass %	Lattice Parameters, Ǻ	CSR Size, nm
Cr_x_Ni_y_-TiN	TiN_225	70	a = 4.2425	85
	CrNi_229	30	a = 2.8877	55

**Table 5:** Results of the energy dispersive analysis of the local areas in the structure of the materials obtained by direct laser deposition from CrNi-TiN SHS composites.

Point No. (+)	Detected Elements, at. %			
	Ti	N	Cr	Ni
1	68.5	19.3	9.3	2.9
2	8.6	2.8	59.3	29.3
3	63.6	21.2	11.4	3.8
4	15.7	5.2	52.3	26.8
5	17.2	5.7	51.4	25.7
6 *	62.8	20.9	10.8	5.5

* The areas of elemental analysis in Figure 5c.

## Data Availability

Data sharing is not applicable for this article.
